# Clinical Utility and Limitations of Traditional Risk Scores (EuroSCORE, EuroSCORE II, and STS-PROM) in Patients Undergoing TAVI: A Narrative Review

**DOI:** 10.3390/jcm15114113

**Published:** 2026-05-26

**Authors:** Filip Klausa, Natalia Świątoniowska-Lonc, Anna Skotny, Marek A. Mak, Agnieszka Wysokińska-Kordybach, Jacek Skiba, Krzysztof Ściborski, Waldemar Banasiak, Adrian Doroszko

**Affiliations:** 1 Department of Cardiac Surgery, Centre for Heart Diseases, 4th Military Hospital, 50-981 Wroclaw, Poland; mmak@4wsk.pl (M.A.M.); jskiba@4wsk.pl (J.S.); 2 Department of Cardiology, Centre for Heart Diseases, 4th Military Hospital, 50-981 Wroclaw, Poland; nswiatoniowska-lonc@4wsk.pl (N.Ś.-L.); awysokinska-kordybach@4wsk.pl (A.W.-K.); krzysztof.sciborski@pwr.edu.pl (K.Ś.); waldemar.banasiak@pwr.edu.pl (W.B.); adrian.doroszko@pwr.edu.pl (A.D.); 3 Clinical Research Support Center, 4th Military Hospital, 50-981 Wroclaw, Poland; askotny@4wsk.pl; 4 Harvard T.H. Chan School of Public Health—Executive and Continuing Education, Harvard University, Boston, MA 02115, USA; 5 Clinical Department of Cardiology, Faculty of Medicine, Wroclaw University of Science and Technology, 50-981 Wroclaw, Poland

**Keywords:** transcatheter aortic valve replacement, risk assessment, heart valve prosthesis implantation, frailty

## Abstract

The rapid evolution of structural heart interventions, particularly transcatheter aortic valve implantation (TAVI), transcatheter edge-to-edge repair (TEER), and hybrid procedures, has significantly expanded treatment options for elderly, frail, and multimorbid patients previously considered high risk or inoperable. However, perioperative risk stratification in this population remains challenging. Traditional risk scores such as EuroSCORE, EuroSCORE II, STS-PROM, CHA_2_DS_2_-VASc, and HAS-BLED were developed and validated primarily in cohorts undergoing conventional open-heart surgery (CABG and surgical valve replacement) more than 15–25 years ago. This narrative review critically evaluates the performance and limitations of these classical models in contemporary populations undergoing modern structural cardiac interventions. Evidence from registries and meta-analyses indicates only moderate discriminatory ability and systematic calibration errors. EuroSCORE II and STS-PROM frequently overestimate risk in low- and intermediate-risk patients while underestimating it in high-risk and frail individuals, particularly regarding neurological, renal complications, and prolonged hospitalization. Similar limitations apply to CHA_2_DS_2_-VASc and HAS-BLED when used beyond their original scope in the peri-procedural setting of TAVI/TEER. The review highlights the growing role of frailty assessment, procedure-specific variables, and machine learning algorithms, which demonstrate superior predictive performance compared to conventional scores. Until dedicated, regularly updated risk models based on large TAVI/TEER registries become available, traditional scores should be used only as supportive tools within multidisciplinary Heart Team discussions that incorporate individual frailty, quality of life, and patient preferences.

## 1. Introduction

The early 21st century also marked a period of rapid advancement in cardiac surgery and interventional cardiology. Over the course of more than 20 years, we have transitioned from open-heart surgeries—such as coronary artery bypass grafting (CABG) and surgical aortic valve replacement (SAVR)—to percutaneous procedures characterized by a significantly lower degree of invasiveness, such as transcatheter aortic valve implantation (TAVI), transcatheter edge-to-edge repair (TEER), tricuspid valve procedures, and hybrid procedures [1]. At the same time, cardiac surgery has also evolved, albeit in a less spectacular but equally important manner for patient safety. Examples include minimally invasive surgical aortic valve replacement (mini-SAVR), robotic-assisted mitral valve repair, and enhanced recovery protocols, which have improved outcomes and reduced the invasiveness of surgical approaches. This makes the comparison between percutaneous and surgical strategies increasingly nuanced and procedure-specific. This advancement has primarily made it possible to improve the safety of procedures for elderly patients, those with comorbidities, and those with frailty—a group of patients who were previously often considered high risk or even deemed inoperable [2]. Predictive models for perioperative risk are becoming increasingly important in treatment planning. Thanks to advances in artificial intelligence (AI) and machine learning (ML), they achieve significantly higher accuracy than traditional scales, as confirmed by a 2025 meta-analysis of TAVI procedures [3,4].

In routine clinical practice, the EuroSCORE scale (developed in 1999 based on data from 1995) and its updated version, EuroSCORE II (2012), as well as the American STS Predicted Risk of Mortality (STS-PROM, 2008), are still the most commonly used [1,5,6]. Although the era of cardiac surgery risk scores is no longer contemporary, the historical idea of using such scores was revolutionary, as it allowed the development of a common metric for risk stratification across different centers and populations. For patients with concomitant atrial fibrillation, the CHA_2_DS_2_-VASc and HAS-BLED scales are used [7]. It should be noted, however, that these models were developed and validated in a population undergoing conventional open-heart surgery—primarily CABG and valve surgeries. This is significant because the scales were developed for a population in which the average age, multiple comorbidities, and nature of procedures differed from those in today’s clinical reality [1,8]. This leads to significant limitations in the application of these tools in patients eligible for transcatheter structural interventions [9,10].

Currently, these scales are routinely used in a much broader range of cases—including TAVI in geriatric patients with multiple comorbidities, in hybrid procedures, and in patients with frailty [10]. As a result, we find ourselves in a situation where these scales are not only being applied to patient groups other than those for which they were originally designed, but, more importantly, they lack validation in these new populations. This raises questions about the appropriateness of their use in this context [10]. Meta-analyses have shown that in TAVI populations, both EuroSCORE II and STS-PROM achieve a c-statistic (AUC—area under the curve) not exceeding 0.70 for 30-day mortality. Values in this range are generally considered poor to fair, indicating only modest discriminatory ability [11,12]. In fact, these models underestimate in-hospital and 30-day mortality in geriatric patients, while in younger individuals they overestimate the risk of death [13,14].

A similar issue also applies to surgical valve procedures—surgical aortic valve replacement (SAVR) and mitral valve repair (MVR). In this case as well, the use of EuroSCORE II is not optimal, as the scale was not designed with modern cardiac surgery in mind, where perioperative mortality is lower [14,15]. In contrast, the CHA_2_DS_2_-VASc and HAS-BLED scales are currently used in a much broader context than originally intended, suggesting the need for their re-evaluation in the context of structural interventions [16,17].

As a result, we find ourselves in a paradoxical situation: we have extremely effective therapeutic methods at our disposal, but due to the failure to update predictive models, cardiac structural interventions are subject to errors during patient selection—specifically, overestimating or underestimating treatment outcomes. Without taking these limitations into account, the available tools cannot be fully utilized.

The aim of this narrative review is to critically evaluate the utility and limitations of traditional perioperative risk scores (EuroSCORE, EuroSCORE II, STS-PROM, CHA_2_DS_2_-VASc, HAS-BLED) in patient populations undergoing modern structural cardiac interventions.

## 2. Search Strategy

A structured literature search was conducted in the PubMed/MEDLINE, Embase, and Cochrane Library databases from January 2010 to 31 January 2026. The strategy was based on a combination of MeSH/Emtree terms and keywords from three thematic categories:

- Structural cardiac interventions (TAVI/TAVR, TEER, MitraClip, PASCAL, TriClip, hybrid procedures, SAVR, MVR),
- Risk models (EuroSCORE, EuroSCORE II, STS-PROM, CHA_2_DS_2_-VASc, HAS-BLED, risk score*, risk model*),
- Clinical outcomes (mortality, 30-day mortality, in-hospital mortality, stroke, acute kidney injury, outcome*, prediction, AUC, calibration).

Inclusion criteria:

- Observational studies, registries, or meta-analyses involving adults undergoing structural cardiac interventions,
- Evaluation of classical risk models or comparison with new tools,
- Reporting of predictive ability (AUC/c-statistic, calibration, O/E ratio) or clinical endpoints.

Exclusion criteria: case reports, series of <20 patients, animal/in vitro studies, publications without numerical data on risk or outcomes. Selection process: initial assessment of titles and abstracts, followed by full-text review. Due to the narrative nature of the review, no formal risk of bias assessment or quantitative meta-analysis was performed. The synthesis was narrative in nature, with an emphasis on key validation studies from the past 10–15 years.

## 3. Most Commonly Used Risk Scales—Description and Original Cohorts

Several predictive models for perioperative risk are used in routine clinical practice. One of the most important models is EuroSCORE, which was developed between 1995 and 1999 based on data collected from 19,030 patients who underwent cardiac surgery at 128 different centers in Europe [18,19]. The model was created primarily based on valve surgeries (approx. 30%) and CABG (approx. 60%) and included 17 clinical variables [18,19]. During validation, the model achieved discrimination power very good (AUC 0.79) and the calibration of the model was satisfactory [18,19].

The next iteration is EuroSCORE II, which was developed based on data collected from 22,381 patients who underwent cardiac surgery between 2010 and 2011 [1]. The model took into account demographic changes and, importantly, a decline in perioperative mortality. The number of variables in the model increased to 18—some were removed, and new ones were introduced. This resulted in a higher AUC value 0.81 (very good discrimination) which represented significant progress compared to the original EuroSCORE [1,8].

Another scale used in routine clinical practice is the STS-PROM, which was developed based on data from over 774,000 patients—separately for valve surgery and CABG [5,6]. The data were collected in the United States between 2002 and 2006. The proposed model was highly complex; depending on the version, the number of variables ranged from 30 to 40. In the original validation, the AUC/c-statistics for 30-day mortality was 0.81 [5,6].

The CHA_2_DS_2_-VASc scale, used to assess stroke risk in patients with atrial fibrillation, was developed in 2010 as an extension of the earlier 2006 Birmingham/NICE scheme. The modifications introduced at that time involved the addition of new risk factors, which were intended to improve the stratification of patients into lower-risk groups. Data collected from 1084 patients in 2003–2004 from the Euro Heart Survey on Atrial Fibrillation were used to validate the model. However, compared to the CHADS_2_ scale, these changes did not result in a significant improvement in the AUC values for the annual risk of stroke and systemic embolism in this development cohort [20].

That same year, the HAS-BLED scale was introduced for assessing the risk of bleeding in patients with atrial fibrillation. It was developed based on a database of 3978 patients from the European Euro Heart Survey on AF registry. The AUC/c-statistic for the annual risk of major bleeding was 0.72, indicating fair discriminatory ability [21].

It should be noted, however, that neither CHA_2_DS_2_-VASc nor HAS-BLED were developed as tools for assessing perioperative risk in terms of mortality, eligibility for surgery, or serious complications following cardiac surgery or transcatheter interventions. Their original purpose was long-term risk stratification for ischemic stroke (CHA_2_DS_2_-VASc) and bleeding (HAS-BLED). They are mentioned in this review because they are currently also used in the perioperative period for patients undergoing TAVI. Consequently, this confirms the discussed issue of using tools outside their validated scope.

Table 1 summarizes the key characteristics and original performance of these risk scores.

## 4. Validation of Risk Scales in Contemporary Patient Populations

It is important to distinguish between two fundamental aspects of risk model performance: discrimination and calibration. Discrimination refers to a model’s ability to correctly differentiate between patients who will and will not experience the outcome of interest. This is typically quantified by the c-statistic (area under the receiver operating characteristic curve, AUC). In contrast, calibration measures the agreement between the model’s predicted probabilities and the actual observed event rates. A model may demonstrate acceptable discrimination yet exhibit poor calibration—and vice-versa. Both metrics are essential for clinical utility, but many previous studies and reviews have focused primarily on discrimination while underappreciating the often more critical issue of calibration.

These limitations of traditional risk models become particularly evident when applied to contemporary patient populations. Advances in medicine and demographic changes have led to shifts in the demographic structure of highly developed countries over the past few decades—we are observing an increase in the average age and an aging population. This phenomenon has also influenced the characteristics of patient populations. As a result, the largest increase in the number of structural cardiac procedures over the past decade has been in TAVI. This procedure is performed on elderly patients (often > 80 years old) with multiple comorbidities and frailty. Between 1990 and 2000, a significant proportion of patients with this profile were considered inoperable, which is no longer the case today. Given such a significant change in the characteristics of the population, it has become necessary to revalidate the risk scales that were originally developed for a younger group of patients.

EuroSCORE II and STS-PROM remain among the most commonly used tools for pre-TAVI risk stratification. Meta-analyses and registries conducted in recent years show that both scales currently have moderate utility. In the overall TAVI population, both the dedicated TVT risk score and STS-PROM showed poor prediction of 30-day mortality (c-statistic: TVT 0.68 vs. STS 0.64) [10]. Similarly, other studies have reported low discriminatory ability for both scales, with AUC values ranging from 0.60 to 0.68 for 30-day mortality [8,22]. Furthermore, systematic calibration errors are evident. In low- and intermediate-risk patients, EuroSCORE II overestimates the actual risk of perioperative mortality; STS-PROM performs better, though it slightly underestimates the risk [10,13,15]. In contrast, in high-risk patients or those with frailty, both models underestimate the risk, particularly regarding neurological and renal complications or prolonged hospitalization [3,16]. A tendency to overestimate risk has also been observed in patients with severe heart failure and concomitant atrial fibrillation [8,22].

In patients with atrial fibrillation undergoing cardiac surgery, EuroSCORE II demonstrates good discriminatory power, but its calibration remains unsatisfactory. The model consistently overestimates the risk of in-hospital mortality compared to actual outcomes, reflecting a broader trend where traditional risk scores fail to accurately predict perioperative risk in contemporary populations [14].

Recent studies confirm the need to develop patient-specific models for patients undergoing TAVI. In an analysis of 2256 transcatheter aortic valve implantation procedures, the PRE-TAVR model was developed, which accounts for procedure-specific factors, including advanced heart failure (NYHA Class IV), advanced COPD, atrial fibrillation, prior stroke, cancer, and selected laboratory and hemodynamic parameters. This model achieved significantly better discriminatory power for annual mortality (c-statistic 0.770) than EuroSCORE II (0.645) and the STS/ACC TAVR score (0.714), while using fewer variables [16].

In hybrid procedures (e.g., TAVI + CABG or hybrid coronary revascularization), data on the validation of traditional risk scores are particularly limited. A meta-analysis by Sardar and colleagues compared clinical outcomes of hybrid coronary revascularization versus conventional CABG, demonstrating comparable or improved perioperative outcomes with the hybrid approach [23]. However, evidence validating the predictive accuracy of EuroSCORE II and STS-PROM in this specific population remains scarce. It can therefore be hypothesized that these models may demonstrate lower predictive ability than in the case of isolated CABG or TAVI, as they do not account for the cumulative risk resulting from two different therapeutic strategies, prolonged procedure time, and the specific nature of vascular access—factors that could contribute to poorer calibration and an increased risk of inaccurate estimation of perioperative complications.

The limitations of traditional models are most evident in geriatric populations with frailty and comorbidities. Frailty constitutes the most critical unmeasured confounder in conventional perioperative risk scores, significantly limiting their accuracy in contemporary TAVI and TEER populations. EuroSCORE II and STS-PROM systematically lose accuracy as the degree of frailty and the number of comorbidities increase, leading to errors in patient eligibility for treatment and in the risk communication process [24,25].

## 5. Evidence Supporting the Need to Modify or Recalibrate the Models

Despite the widespread use of EuroSCORE II, STS-PROM, CHA_2_DS_2_-VASc, and HAS-BLED in clinical practice, a growing body of evidence suggests that these models require urgent modification, recalibration, or even replacement with newer tools in the context of modern structural cardiac interventions.

The strongest arguments in favor of the need for change come from calibration analyses in the TAVI population. In the low- and intermediate-risk patient groups (STS-PROM < 8%), EuroSCORE II consistently overestimates actual 30-day mortality, which may lead to the unnecessary exclusion of patients from transcatheter therapy or discourage them from undergoing the procedure [10,12]. Conversely, in high-risk patients and those with frailty, these models underestimate the risk of neurological and renal complications as well as prolonged hospitalization [13,15].

Poor calibration has significant clinical implications: it leads to excessive caution in selecting low-risk patients for TAVI, inadequate protection of high-risk patients, and difficulties in comparing outcomes across different centers.

These limitations have necessitated modifications to existing models or even the creation of new tools. An example of this is the PRE-TAVR model, which incorporates additional variables specific to TAVI [16]. The AUC of this model is 0.77, which is higher than the corresponding AUC values for the older EuroSCORE II and STS/ACC TVT models, which are 0.645 and 0.714, respectively [16].

With the advancement of artificial intelligence, machine learning (ML)-based models are also playing an increasingly important role. Recent meta-analyses from 2025 indicate that machine learning models achieve a c-statistic of 0.78 for mortality following TAVI, outperforming traditional risk scores [3,4]. A systematic review conducted in accordance with the PRISMA guidelines by Liscano et al. provides some very interesting insights in this context [26]. The authors analyzed data on over 533,000 patients and demonstrated that the use of machine learning-based models (including meta-learning) can significantly increase predictive value compared to conventional risk scales. F. Tsakirian et al. conducted a literature review, based on which they suggested that the use of ML models could help revolutionize and optimize the decision-making process at each stage of the TAVI procedure [27]. These conclusions were based on an analysis of 51 articles selected from among 7177 publications identified during the screening process. Both studies clearly indicate that the integration of ML into the TAVI workflow may soon become a routine tool for Heart Teams planning and performing these procedures. The use of ML enables faster analysis of larger datasets and the assessment of nonlinear interactions between variables, which is extremely difficult to achieve in classical regression models.

The CHA_2_DS_2_-VASc and HAS-BLED scales are established tools for stroke and bleeding risk assessment in patients with atrial fibrillation, and their association with frailty and adverse outcomes has been demonstrated, among others, in the TREAT-AF study [7]. However, their utility in the setting of transcatheter valve procedures appears more limited. Veulemans et al. found no significant association between baseline CHA_2_DS_2_-VASc and HAS-BLED scores and 30-day mortality after TAVI [28]. Similarly, a recent analysis showed that while machine learning models performed well, traditional CHA_2_DS_2_-VASc and HAS-BLED scores had only modest predictive ability for adverse events in TAVI patients with atrial fibrillation [29]. These findings suggest that static pre-procedural values of these scores may lose predictive power in the post-TAVI/TEER period, supporting the need for dynamic risk reassessment in this population.

Traditional risk models serve as an important reference point; however, their routine use in the era of structural cardiac interventions without recalibration or modification leads to systematic clinical errors. There is an urgent need to develop dedicated, up-to-date tools—ideally based on large TAVI/TEER registries and machine learning methods—that better reflect the current clinical reality.

## 6. Discussion

The data presented clearly indicate that traditional perioperative risk scores, such as EuroSCORE II, STS-PROM, CHA_2_DS_2_-VASc, and HAS-BLED, currently have limited clinical utility—due both to changes in procedural techniques and to the evolving patient population to which they are applied. Unfortunately, these tools are still used in routine clinical practice in TAVI, TEER, and hybrid procedure populations, as well as in geriatric patients with frailty. Using them in their unmodified form leads to systematic prediction errors [10,11].

The main problem is not so much the decrease in AUC (discriminatory power) as it is, above all, poor calibration. Moreover, traditional risk models primarily focus on mortality prediction and may fail to adequately capture other clinically relevant peri-procedural complications, such as myocardial injury and infarction, which have emerged as important determinants of postoperative outcomes in the era of structural heart interventions [30]. EuroSCORE II consistently overestimates the risk of mortality in low- and intermediate-risk patients, which may lead to the unjustified exclusion of patients from percutaneous therapy or cause undue anxiety in the patient [12,13]. Conversely, in high-risk patients and those with advanced frailty, these models underestimate the risk of complications, particularly neurological and renal ones [15,16]. STS-PROM performs slightly better but is also not free from these limitations [12]. The CHA_2_DS_2_-VASc and HAS-BLED scales, originally developed to assess the risk of stroke and bleeding in atrial fibrillation, are currently used outside their original context, which further reduces their predictive value in the perioperative period [7,17].

The clinical implications of these shortcomings are significant: incorrect patient selection for treatment strategies, suboptimal patient counseling regarding risks, and difficulties in objectively comparing outcomes across centers and countries. While these traditional scores remain a cornerstone of TAVI trial design and patient stratification based on surgical risk, their performance in contemporary practice is increasingly questioned. The surgical mindset is different from the cardiology interventional mindset—what constitutes an acceptable risk, how frailty is weighed, and which complications are prioritized often differ between specialties. At a time when perioperative mortality in TAVI is falling below 1–2% in many registries and is comparable to that of the general population in low-risk patients, the use of tools developed 15–25 years ago is becoming increasingly anachronistic [31,32].

Currently available approaches—recalibrating existing models, incorporating variables specific to structural procedures (frailty, CT data, biomarkers), and developing machine learning-based algorithms—appear to be the most promising direction. Meta-analyses from 2025 consistently show a clear advantage of ML models (pooled AUC 0.78–0.85) over traditional scales [3,4]. However, these new tools require prospective external validation across different populations and healthcare systems before they can be implemented into daily practice.

Until dedicated, regularly updated predictive models are developed, clinicians should treat EuroSCORE II and STS-PROM scores as indicative rather than definitive. The final treatment decision must result from a multidisciplinary Heart Team discussion, taking into account not only the score but also an individual assessment of frailty, quality of life, the patient’s expectations, and the center’s experience [9].

It should be emphasized that the limitations of traditional perioperative risk scales in the population of patients undergoing structural cardiac interventions are a frequent topic in previous literature reviews. This highlights the scale of the problem and, at the same time, confirms that this issue still requires an optimal solution. This review provides an updated synthesis of the literature from a clinical perspective. It pays particular attention to machine learning-based models, which may revolutionize risk stratification in the coming years.

In the context of TAVI, newer, dedicated models such as FRANCE-2, UK-TAVI, and STS/ACC TVT generally demonstrate better discriminatory power than EuroSCORE II or STS-PROM [10,33,34]. An example of added value is the inclusion of frailty, which in the UK-TAVI model proved to be a significant, independent predictor of mortality following TAVI [34].

For TEER, the MitraScore and MITRALITY models demonstrate promising predictive value for mortality, while TRI-SCORE (originally developed for mitral valve surgery) is increasingly used in transcatheter mitral valve interventions, offering better prognostic value than traditional scales [35,36,37]. While these models obviously have their limitations (including imperfect calibration and limited external validation), they offer a significant new perspective by accounting for procedure-specific variables and better adapting to patient populations undergoing structural interventions.

Data on hybrid procedures remain limited, which represents a significant gap in the current body of knowledge.

This review aims to highlight these inconsistencies and potential directions for future research, going beyond the merely confirmatory nature of the conclusions drawn in other studies and offering a set of guidelines for Heart Teams in routine clinical practice. Of course, it should be noted that this is a narrative review rather than one conducted in accordance with the PRISMA guidelines, which may influence the subjectivity of the assessment and selection of studies resulting from the authors’ clinical experiences.

## 7. Conclusions

Traditional perioperative risk scores (EuroSCORE, EuroSCORE II, STS-PROM, CHA_2_DS_2_-VASc, and HAS-BLED) were developed in a clinical context vastly different from the one we face today. Originally intended primarily for patients undergoing CABG and surgical valve procedures, today they are routinely used in geriatric populations with frailty and multimorbidity, as well as in percutaneous and hybrid procedures (TAVI, TEER, tricuspid valve procedures). This review shows that these models have only moderate predictive ability (as reported in meta-analyses, pooled AUC ranges from 0.62 to 0.70) and systematic calibration errors—EuroSCORE II most often overestimates risk in low- and intermediate-risk patients, while underestimating it in high-risk patients and those with frailty. Similar limitations apply to STS-PROM and the CHA_2_DS_2_-VASc and HAS-BLED scales in the peri-procedural context. Given the rapid advancement of structural cardiac interventions, continued reliance solely on these tools is unjustified and may lead to errors in patient selection, suboptimal risk communication, and difficulties in comparing outcomes across centers.

In conclusion, traditional perioperative risk scores should no longer be used as standalone tools for risk stratification or patient selection in individuals undergoing transcatheter structural heart interventions. Their role should be limited to supportive instruments within multidisciplinary Heart Team discussions, which must integrate frailty assessment, procedure-specific factors, quality of life, and patient preferences. In the near future, machine learning-based models are expected to play a central role in this field. Until then, reliance on outdated legacy scores as the primary decision-making tool is no longer justified.

## 8. Limitations

This study is a narrative review, which entails certain methodological limitations. No formal assessment of risk of bias was conducted, nor was a quantitative synthesis of results in the form of a meta-analysis performed. Although the selection of sources was based on a structured search strategy, it may have been subject to the subjective selection typical of this type of study. Furthermore, due to the publication’s length constraints, a detailed analysis of all available predictive models (e.g., FRANCE-2, STS/ACC TVT) was not included, nor was a systematic comparison of predictive ability presented for each of the endpoints discussed. Despite these limitations, the review provides an up-to-date and practical synthesis of key issues related to the use of traditional risk scales in the era of structural cardiac interventions.

## 9. Practical Recommendations

- EuroSCORE II and STS-PROM scores should be considered indicative rather than definitive.
- Treatment decisions must be based on a multidisciplinary Heart Team discussion, taking into account frailty assessment, quality of life, and patient preferences.
- There is an urgent need to develop and validate new, dedicated risk models for structural procedures, ideally based on large registries and machine learning methods.
- Until such tools are introduced, it is recommended to use local recalibration or to add TAVI/TEER-specific variables (frailty, CT data, biomarkers).

## Figures and Tables

**Table 1:** Comparison of the most commonly used perioperative risk scores—original development and validation characteristics.

Risk Score	Year	Original Cohort	No. of Variables	Main Procedures	c-Statistic (AUC)	Discriminatory Ability
EuroSCORE	1999	19,030 (Europe)	17	CABG (60%), valve (30%)	0.78–0.82	Good
EuroSCORE II	2012	22,381 (Europe)	18	Broad cardiac surgery	0.81–0.85	Good
STS-PROM	2008	>774,000 procedures, USA	30–40 lub 20+	CABG and isolated valve surgery	0.80–0.82	Good
CHA_2_DS_2_-VASc	2010	1084 patients, Euro Heart Survey AF	9	Atrial fibrillation	0.61	Poor to fair
HAS-BLED	2010	3978 Euro Heart Survey AF	9	Atrial fibrillation	0.72	Fair

## Data Availability

No new data were created or analyzed in this study.

## Abbreviations

The following abbreviations are used in this manuscript:

AF	Atrial Fibrillation
AUC	Area Under the Curve (c-statistic)
CABG	Coronary Artery Bypass Grafting
CHA_2_DS_2_-VASc	Congestive heart failure, Hypertension, Age ≥ 75 (doubled), Diabetes, Stroke (doubled), Vascular disease, Age 65–74, Sex category (female)
COPD	Chronic Obstructive Pulmonary Disease
HAS-BLED	Hypertension, Abnormal renal/liver function, Stroke, Bleeding history or predisposition, Labile INR, Elderly (>65), Drugs/alcohol concomitantly
ML	Machine Learning
MVR	Mitral Valve Repair/Replacement
NYHA	New York Heart Association
O/E	Observed/Expected ratio
PRE-TAVR	Patient Risk Evaluation for Transcatheter Aortic Valve Replacement
SAVR	Surgical Aortic Valve Replacement
STS-PROM	Society of Thoracic Surgeons Predicted Risk of Mortality
TAVI (TAVR)	Transcatheter Aortic Valve Implantation (Replacement)
TEER	Transcatheter Edge-to-Edge Repair
TVT	Transcatheter Valve Therapy (registry)
